# Revision of subjects of the Korean Optometrist Licensing Examination suggested by optometrists

**DOI:** 10.3352/jeehp.2012.9.5

**Published:** 2012-03-14

**Authors:** Won Jin Lee, Sung Soo Kang, Ok-Jin Lee, Sang-Chul Park, Seung-Won Lee, Young-Ki Jeon

**Affiliations:** 1 Department of Ophthalmic Optics, Daegu Polytechnic College, Daegu, Korea.; 2 Department of Optometry and Vision Science, Dongnam Health College, Suwon, Korea.; 3 Department of Ophthalmic Optics, Busan College of Information Technology, Busan, Korea.; 4 Department of Ophthalmic Optics, Kundong University, Andong, Korea.

Since the introduction of the optometrist licensing system to the medical technician law in 1987, the first Korean Optometrist Licensing Examination was carried out on October 22, 1989. After that, there have been a variety of environmental changes. An optometry major was newly created in a number of colleges. There was a change of curriculum from a 2-year program to a 3-year program, and eventually to a 4-year one. As a result, a profound approach to subject revision of the Korean Optometrist Licensing Examination became necessary from the perspective of optometrist manpower and its management system.

Before these changes, the Korean Optometrist Licensing Examination consisted of four written examination categories: optometry, ophthalmic optics, ophthalmology, and medical health law. There was another separate performance examination. The detailed subjects covered by the written examination were as follows: ophthalmic optics, geometric optics, physical optics, ophthalmic optics instruments, prescribing and dispensing of eyeglasses, introduction to eyeglasses, glasses materials, ophthalmic anatomy, ophthalmic physiology, ophthalmic diseases, and abnormal ophthalmic function.

The improvements to the Korean Optometrist Licensing Examination were focused on specific subjects and items. In 1988 we published the results of a job analysis through a questionnaire survey on the optometrist's job from 813 optometrists and 89 ophthalmic optic professors. It showed that optometric dispensing was the most important among their essential tasks and educational needs. Meanwhile, medical health law and business management was ranked least important out of six duty categories of optometrists, specifically, preliminary examination for visual acuity, visual test and refraction, prescription, optometric dispensing, follow-up, and medical health law and management [1]. After the analysis of the survey results, the findings were opened to be discussed by optometry professionals. The consensus of opinions from public hearings and workshops with ophthalmic optics professors were reflected in the suggestion of subjects. Revision of the subjects of the Korean Optometrist Licensing Examination was suggested as follows:

First, the examination subject entitled '*introduction to eyeglasses*' should be removed and the corresponding contents included in other specific subjects. Second, content on contact lens included in '*glasses materials*' should be allocated to a new subject entitled '*eyesight correction materials*' composed of the subsections '*contact lens materials*' and '*glasses frame and lens materials*'. Third, the subject 'optometry' containing refraction examination and stereovision function tests should be added and be emphasized.

Some of these changes are still under consideration by the National Health Licensing Examination Board of Korea. Further discussion is also necessary for the revision of the performance test after considering the availability of space and instruments in each college as well as the building of the National Health Licensing Examination Board.
